# To What Extent Are Cryopreserved Sperm and Testicular Biopsy Samples Used in Assisted Reproduction?

**Published:** 2018

**Authors:** Bernd Rosenbusch

**Affiliations:** - Department of Gynaecology and Obstetrics, Ulm University, Ulm, Germany

**Keywords:** Assisted reproduction, Cryopreservation, Male fertility preservation, Spermatozoa, Testicular biopsy

## Abstract

**Background:**

Testicular biopsies and ejaculated spermatozoa are routinely cryopreserved in many units but the fate of these samples has not provoked large interest. This prompted us to review our data accumulated during a period of 20 years (1997 to 2016).

**Methods:**

For patients with biopsies (group 1) or ejaculated spermatozoa (group 2), an attempt was made to evaluate whether the samples stored, had been discarded with the patient’s consent or because the patient had died, or whether they had been transported to another laboratory. In each of these categories, a previous use in our program of assisted reproduction was assessed.

**Results:**

The total utilization rate in group 1 (n=95) was 53.7% and only 5.48% in group 2 (n=365). In both groups, deceased patients had not previously used their cryopreserved samples. In detail, the utilization rates for still banked, discarded and transferred samples were 84.2%, 50% and 27.3%, respectively in group 1 and 2.88%, 10.4% and 10%, respectively in group 2.

**Conclusion:**

The exact reasons for the low utilization rates of cryopreserved male gametes remain to be explored. A closer contact between sperm banking units and patients might be useful to discuss the need for further storage of the probes, their possible disposal or the prospects when a specific use for assisted reproduction is intended.

## Introduction

The cryopreservation of gametes, fertilized oocytes and cleaved embryos is an important component of assisted reproductive technology (ART). Male patients can store spermatozoa if they have psychologic or occupational problems to collect a fresh sample at a certain date. Azoospermic patients can undergo a testicular biopsy with subsequent cryopreservation of the samples if viable sperm are present. Another essential reason for banking spermatozoa is fertility preservation for men affected by different malignant diseases because the treatments like chemotherapy and radiotherapy can severely impair spermatogenesis temporarily or permanently.

It is known that the fertility potential in cancer patients is often decreased before treatment (1) and that the freezing/thawing process will further compromise the quality of the specimens. Thus, it should be expected that a high rate of these patients will be candidates for ART because with intracytoplasmic sperm injection (ICSI) as the ultimate means, an oocyte needs only one viable spermatozoon for fertilization. Despite this unique opportunity, the reported utilization rates are surprisingly low (2, 3). This prompted us to assess the fate of cryopreserved testicular biopsies and sperm samples in our unit, considering a period of 20 years (1997 to 2016).

## Methods

Infertility patients revealing azoospermia underwent a testicular biopsy at the Urological Department of Ulm University. The samples were frozen and stored in the laboratory of our ART center if the presence of spermatozoa had been confirmed (Group 1). The second group consisted of men referred to our unit for sperm banking before therapy of various diseases, mostly Hodgkin‘s lymphoma or testicular cancer. A few patients requested temporary cryopreservation because they were not present on the day of follicular aspiration. They were excluded from the present analysis.

Testicular tissue and ejaculated spermatozoa were frozen after addition of a commercially available cryoprotectant according to the instructions of the supplier. Currently, SpermStore GM501) obtained from GYNEMED (Lensahn, Germany) was used for our purpose. Nunc^TM^ cryo vials were used for cryopreservation of testicular tissue whereas aliquots of the ejaculate were transferred to high security plastic straws (HS-set, Minitüb GmbH, Tiefenbach, Germany). Initially, the samples were cooled in vapor phase nitrogen for 30 *min* and then plunged into liquid nitrogen for storage. Since 2010, a freezing program has been applied that lowers the temperature in four steps to −190°*C*. Pre-freeze sperm concentration and motility have always been recorded. Ejaculates showing a low sperm density but large volume were concentrated by centrifugation. Samples containing a lot of debris, round cells and dead spermatozoa were cleaned using a two-step density gradient centrifugation. Our intention was to freeze 12 straws of ejaculate and at least five tubes with testicular tissue, but some cases with very low sperm concentration or small testicular size were seen where these numbers had to be reduced to two straws and two tubes, respectively.

## Results

The data of 460 men have been reviewed, of which 95 belonged to group 1 with a mean age of 35.47 years (range: 24–61 years). Group 2 comprised 365 men with a mean age of 28.23 years (range: 13–59 years). The total utilization rate of cryopreserved samples was 53.7% in group 1 and 5.48% in group 2, respectively. Table 1 depicts how many patients have used their discarded or still banked specimens. Moreover, it is shown that none of the deceased patients from both groups had previously requested treatment by assisted reproduction. The utilization rate of samples that were later transferred to another center was higher in group 1. Only 9 patients (5 in group 1 and 4 in group 2) have used all of their cryopreserved samples. They were included under “discarded” (in the sense of “no longer present”). Insertion of an additional column in table 1 was avoided in view of the low number of cases.

**Table 1.** The fate of cryopreserved testicular biopsies (Group 1) and semen samples (Group 2)

**Classification of patients**	**Total number of patients**	**Utilization of banked samples (%)**	**Utilization of discarded samples (%)**	**Previous utilization by deceased patients (%)**	**Utilization of transferred samples (%)**	**Total utilization rate (%)**
**Group 1**	95	16/19 (84.2)	32/64 (50.0)	0/1 (0.0)	3/11 (27.3)	51/95 (53.7)
**Group 2**	365	6/208 (2.88)	13/125 (10.4)	0/22 (0.0)	1/10 (10.0)	20/365 (5.48)

## Discussion

In 1992, Sanger et al. (4) proposed to revise the existing criteria for pretherapy semen cryobanking because pregnancies could be achieved with intrauterine insemination or conventional *in vitro* fertilization even in cases of low sperm density and motility. Nowadays, the availability of ICSI supports the view that regardless of other abnormalities, any sample containing motile spermatozoa should be frozen (1, 5). However, it has also been noted that sperm motility, survival rate and condition of chromatin are severely affected by cryopreservation that will be no major problem for normal ejaculates but for those showing oligoasthenoteratozoospermia. For these patients, the number of ICSI trials will be limited, stressing the need for the development of improved freezing techniques (6).

The number of samples that should be frozen per patient is another point at issue and only few indications or recommendations are found in the literature. Lass et al. (5) asked the patients to collect a sample every 2–3 days until a total of 12 ampoules were obtained for banking but they admitted that this was not always possible if therapy had to start immediately. As noted above, our purpose was to freeze 12 straws of ejaculate and at least 5 probes of testicular tissue. In cases where less material is available, however, it is our experience that thawed probes can be frozen again for another ICSI trial.

Azoospermia is normally detected during the routine infertility work-up of a couple, *i.e*. when the male partner is requested to deliver semen samples. These men are informed about the possibility of testicular sperm extraction and ICSI combined with cryopreservation of the testicular tissue. Consequently, group 1 had already been confronted with fertility problems and appeared to be willing to pursue the goal of having their own child, using all techniques of modern assisted reproduction. Why, however, does the utilization rate in this group then fail to reach 100%? Since the patients were not obliged to give reasons when they end their contract of cryopreservation, there was only speculation about problems within the partnership up to separation of the couple. Some patients might reject their plan of undergoing ICSI after having been informed about presumably low fertilization rates because the biopsies contained only few spermatozoa. Those who had used their probes at least once might give up ART because of the psychological stress of treatment, financial difficulties or because they were satisfied with one successful trial. Of note, a brief literature search in PubMed using the terms “testicular biopsies”, “cryopreservation”, and “utilization rate” yielded only one hit, reporting a utilization rate of 92% (23/25) for frozen testicular spermatozoa (7).

Group 2 was completely different from group 1 because the majority of these men, particularly the younger ones, had not thought about family planning when they were confronted with the diagnosis of their disease. In this unique stress situation, it was our experience that many opted for semen banking but a decision about using the samples would be postponed for an undefined period. The earliest expected date should be the moment when the disease had been overcome. However, the utilization rates mostly remained below 10% (range: 2.7% to 27.0%) even after more than 20 years (2). Other publications reported values between 4.5% and 10.3% (3, 8, 9) and a recent review indicates a mean rate of 8% (10). Our rate of 5.48% is comparable to these findings. Interestingly, the disposal rates also remained low and the reasons cited often included death of the patient (2).

A variety of reasons has been discussed to explain why men that survived their disease do not use their frozen samples. Male psychology could play a role, *i.e*. men may generally be reluctant to take advantage of health care and they may underestimate the extent to which the disease affects their fertility (2). Further possible reasons include the fear of potential risks and low success rates of ART, the fear of transmitting a genetic predisposition to cancer (11) and the fear of using frozen-thawed gametes because this might affect the health of the child. Of course, there are cases of spontaneous recovery of spermatogenesis and successful natural conception while others definitely decide to have no children. Finally, financial aspects may play a role.

Kelleher et al. (12) concluded from their data that stored semen is mostly used in the first three years whereas the utilization rate after 10 years is extremely low. Moreover, frozen sperm had not been used after 15 years or used successfully after 11 years of storage. Therefore, there should be thought about discarding sperm after 10 years (12). However, some patients are between 15 and 20 years of age when they present for cryopreservation and a limited storage time of 10 years appears too short because even after this period, the individuals might still be too young to think about family planning. In fact, Ragni et al. (11) reported an increase in the cumulative rate of use at 12 years but admitted that due to a low number of patients this topic needs further investigation, particularly extending the length of follow-up.

An important question that remains is how sperm banking units can stimulate or improve their patients’ interest in their cryopreserved probes. Pertinent recommendations have been given, for instance the development of systems to maintain contact with those having sperm stored though they have overcome their disease (2). The fact that, like in the UK, consent must be renewed every 10 years (2) may provide a good opportunity to recall the topic of reproduction. Such a written reminder, possibly in shorter intervals, may include the offer to visit the ART center for a semen analysis to check the present fertility status. On this occasion, the need for further storage or the possibility of disposal of the frozen samples could be discussed. The methods of assisted reproduction, if required, could be explained in depth and a questionnaire could be provided inquiring about the attitude towards ART and the above-mentioned fears of using banked semen. Of course, the establishment of a dedicated counselling service will be necessary (2) but in view of the fact that an increase in the number of patients being referred for sperm banking was noticed, this issue will certainly gain more importance.

## Conclusion

Though sperm banking is nowadays offered by many institutions, information about the fate of stored samples has remained limited. The available data reveal that the utilization rates in ART programs are rather low. Moreover, the present report shows that even testicular biopsies from infertility patients are not completely used. Specifying the reasons for these phenomena and trying to improve the demand for frozen probes are challenging future tasks that will involve an intensified contact between sperm banking units and their patients.
